# Mitochondria-related miR-141-3p contributes to mitochondrial dysfunction in HFD-induced obesity by inhibiting *PTEN*

**DOI:** 10.1038/srep16262

**Published:** 2015-11-09

**Authors:** Juan Ji, Yufeng Qin, Jing Ren, Chuncheng Lu, Rong Wang, Xiuliang Dai, Ran Zhou, Zhenyao Huang, Miaofei Xu, Minjian Chen, Wei Wu, Ling Song, Hongbing Shen, Zhibin Hu, Dengshun Miao, Yankai Xia, Xinru Wang

**Affiliations:** 1 State Key Laboratory of Reproductive Medicine, Institute of Toxicology, Nanjing Medical University, Nanjing 210029, China; 2 Key Laboratory of Modern Toxicology of Ministry of Education, School of Public Health, Nanjing Medical University, Nanjing 210029, China; 3 Department of Epidemiology and Biostatistics and Key Laboratory of Modern Toxicology of Ministry of Education, School of Public Health, Nanjing Medical University, Nanjing, China; 4 Research Center for Bone and Stem Cells, Department of Anatomy, Histology, and Embryology, Nanjing Medical University, Nanjing, China

## Abstract

Mitochondria-related microRNAs (miRNAs) have recently emerged as key regulators of cell metabolism and can modulate mitochondrial fusion and division. In order to investigate the roles of mitochondria-related miRNAs played in obesity, we conducted comprehensive molecular analysis *in vitro* and *in vivo*. Based on high-fat-diet (HFD) induced obese mice, we found that hepatic mitochondrial function was markedly altered. Subsequently, we evaluated the expression levels of selected mitochondria-related miRNAs and found that miR-141-3p was up-regulated strikingly in HFD mice. To further verify the role of miR-141-3p in obesity, we carried out gain-and-loss-of-function study in human HepG2 cells. We found that miR-141-3p could modulate ATP production and induce oxidative stress. Through luciferase report gene assay, we identified that phosphatase and tensin homolog *(PTEN)* was a target of miR-141-3p. Inhibiting *PTEN* could alter the mitochondrial function, too. Our study suggested that mitochondria-related miR-141-3p induced mitochondrial dysfunction by inhibiting *PTEN*.

Obesity, a state of low-grade chronic inflammation^1^, predisposes to various diseases, including nonalcoholic fatty liver disease (NAFLD), type 2 diabetes, cancer and heart disease^2^. The prevalence of obesity is increasing dramatically in both developed and developing countries all over the world^3^. It’s predicted that up to 58% of the world’s adult population will be overweight or obese by 2030^3^. The cause of obesity is the unbalance between energy intake and energy expenditure^4^, in which mitochondrion is involved.

Mitochondrion, the unique intracellular organelle, contains its own circular genome, encoding 13 essential proteins of oxidative phosphorylation (OXPHOS)^5^. Mitochondrion participates in a great number of physiological processes including ATP production, calcium homeostasis, free radical species and β-oxidation of free fatty acids (FFA) production^6^. Through OXPHOS^5^, mitochondrion generates not only ATP for the cell energetic metabolism, but also reactive oxygen species (ROS), the toxic by-products of respiration. Excessive ROS induce oxidative stress^7^, causing damage to cells. The alterations of mitochondrial biogenesis, dynamics and function have been observed in numerous metabolic disorders, including obesity, diabetes and NAFLD^8^.

Recently, mitochondria-related microRNAs (miRNAs), encoded by mitochondria genome or nuclear genome, have been found to affect mitochondrial functions including energy metabolism, mitochondrial dynamics, ROS and electron transport chain (ETC)^7^,^9^. Some mitochondria-related miRNAs have been thoroughly investigated in cancer progression and invasion^7^; however, whether mitochondria-related miRNAs are involved in the aetiology of obesity remains unclear. Thus, it’s worthwhile to investigate the role of mitochondria-related miRNAs in obesity.

In view of the above, we hypothesized that mitochondria-related miRNAs might affect mitochondrial function, cause the disequilibrium of energy metabolism and eventually lead to obesity. To test this hypothesis, we applied high-fat-diet (HFD)-induced obese mice to study the function of mitochondria-related miRNAs. To elucidate the potential mechanism of mitochondria-related miRNAs in obesity, we assessed the mitochondrial function in human hepatocarcinoma cells (HepG2 cells) transfected with mitochondria-related miRNAs. And then, additional experiments about signaling pathway were performed to explore the underlying mechanism related to the observed alteration of mitochondrial function.

## Results

### HFD induced obesity and altered metabolic syndrome

After 5 weeks of high-fat-diet, the HFD mice gained significantly more weight (Fig. S1A). At 16-week, the HFD mice appeared fatter than the standard-fat-diet (SD) mice (Fig. S1B). The daily food intake of HFD mice (Fig. S1C) was significantly higher than the SD mice. Moreover, the body weight (Fig. S1D), Lee’s index (Fig. S1E) of HFD mice were significantly higher than that of SD mice. The levels of serum total cholesterol (TC), high density lipoprotein cholesterol (HDL-C), low density lipoprotein cholesterol (LDL-C) in HFD mice were significantly higher than SD mice (Fig. S2A), indicating that the HFD mice developed glycolipid metabolic disorder. In addition, the higher levels of fasting blood glucose and higher glucose level at 60, 90, 120 min of OGTT of HFD mice (Fig. S2B, C) implied that the HFD mice had impaired glucose intolerance. Similarly, the higher level of insulin (Fig. S2D), HOMA-IR (Fig. S2E) and higher glucose level at 0, 30, 60, 90, 120 min of ITT (Fig. S2F) in the HFD mice suggested that the insulin sensitivity was decreased in the HFD mice. All above results indicated that mice got obesity under HFD.

**Figure 1:**
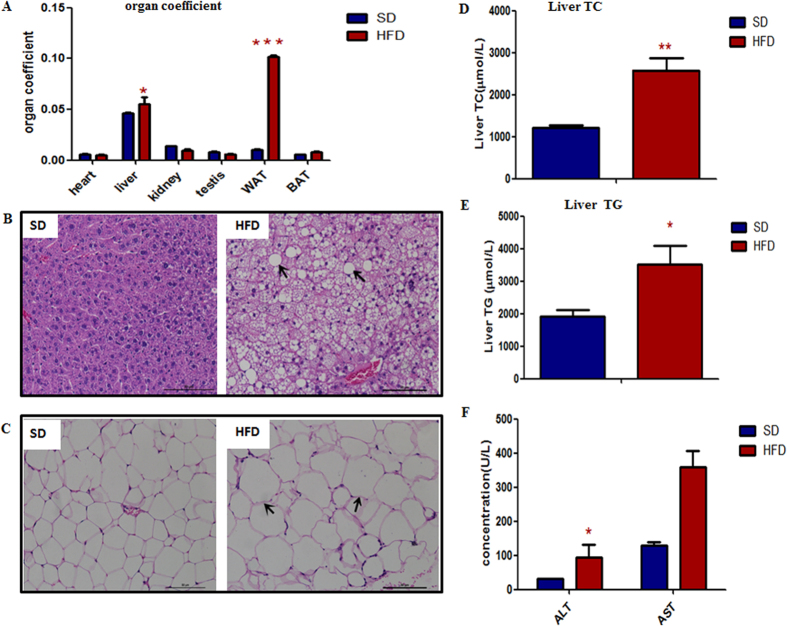
The liver and WAT were obviously abnormal in the 16-week-old HFD mice. (**A**) The organ coefficient of liver and WAT were significantly increased in HFD mice. (**B**,**C**) HE staining of liver and WAT were shown. Scale bar, 50 μm. (**B**) Diffuse and marked fat accumulation in the liver of HFD mice compared with that of SD mice. (**C**) Increased volume of lipocytes in an abdominal fat pad from an HFD mouse compared with that from SD. The level of liver TC (**D**), TG (**E**) and ALT (**F**) were markedly higher in the HFD mice. *P < 0.05,**P < 0.01,***P < 0.001.

The organ coefficients of liver and WAT (white adipose tissue) of HFD mice were significantly higher than SD mice (Fig. 1A). Besides, the marked fat accumulation in the liver and the increased volume of lipocytes in the abdominal fat pad (Fig. 1B,C) were observed in the HFD mice. The levels of liver TC, TG and serum alanine amino transferase (ALT) (Fig. 1D–F) were increased in HFD mice, indicating that the metabolic function of liver was impaired in HFD mice.

### Obesity impaired hepatic mitochondrial function

**Figure 2:**
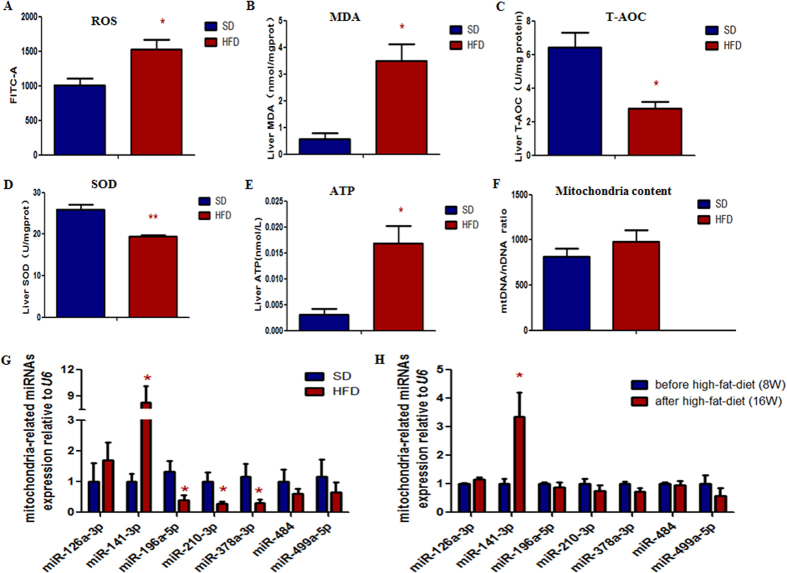
The HFD mice were more vulnerable to oxidative damage and the antioxidant capacity was decreased. (**A**) The DCF average fluorescent intensity was presented in histogram, the ROS production was significantly increased in HFD mice. (**B**) The MDA production was significantly increased in HFD mice. Antioxidant capacity, including T-AOC activities (**C**) and SOD activities (**D**) were measured. Both of them were significantly decreased in HFD mice. ATP production (**E**) was significantly increased in the HFD mice. Mitochondrial content (**F**) were similar between HFD mice and SD mice. The level of mitochondria-related miR-141-3p (**G**) was strikingly increased, and the levels of miR-196a-5p, miR-210-3p and miR-378a-3p were significantly decreased in the liver of 16-week-old HFD mice compared with 16-week-old SD mice. The level of mitochondria-related miR-141-3p (**H**) was markedly increased in the 16-weel-old HFD mice compared with the 8-week-old mice. Each data point represented the mean ± SE from three separate experiments in which treatments were performed in triplicate. *P < 0.05.

To measure the oxidative damage and antioxidant capacity of liver, we detected the reactive oxygen species (ROS) and malonaldehyde (MDA). Interestingly, we found that the production of ROS and MDA (Fig. 2A,B) were strikingly increased in the HFD mice. While the total antioxidant capability (T-AOC) and superoxide dismutase (SOD) activity (Fig. 2C,D) were markedly decreased. To further evaluate the mitochondrial function, we assessed ATP production and found liver ATP production in HFD mice was significantly higher than SD mice (Fig. 2E). To avoid the bias caused by copy numbers of mitochondrial DNA (mtDNA), we also assessed the mtDNA copy number in liver of HFD mice and SD mice. As expected, no significant difference was found between the two groups (Fig. 2F).

### MiR-141-3p highly expressed in HFD mice liver

To further assess the alterations of mitochondria-related miRNAs in obesity, we examined the expression levels of miR-126a-3p, miR-141-3p, miR-196a-5p, miR-210-3p, miR-378a-3p, miR-484 and miR-499a-5p in mice livers. As shown in Fig. 2G, the expression of mitochondria-related miR-141-3p was strikingly increased and miR-196a-5p, miR-210-3p, miR-378a-3p were reduced in the HFD mice. There was no significant difference of miR-126a-3p, miR-484, miR-499a-5p between HFD mice and SD mice. In addition, we also compared the expression pattern of selected miRNAs between 8-week-old mice (before high-fat-diet) and 16-week-old HFD mice (after high-fat-diet). As shown in Fig. 2H, the mitochondria-related miR-141-3p expression level was significantly increased in the16-week-old HFD mice than that in the 8-week-old mice. As to the other mitochondria-related miRNAs, the expression levels were lower than that in 8-week-old mice, although no significant difference was found.

Considering the most strikingly alteration of mitochondria-related miR-141-3p expression level in the mice, miR-141-3p was selected to investigate the potential mechanism involved in hepatic mitochondrial function.

### MiR-141-3p regulates mitochondrial function in HepG2 cells

**Figure 3:**
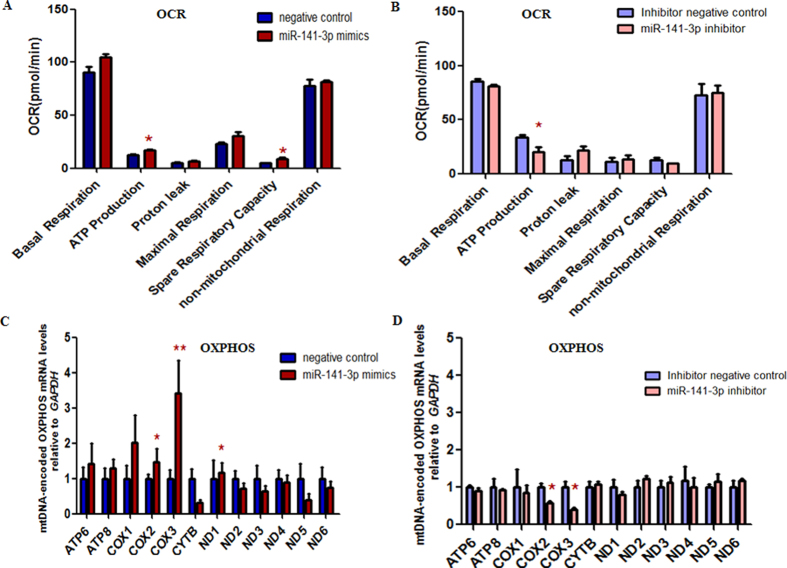
The miR-141-3p may promote mitochondrial respiratory function of HepG2 cells. (**A**,**B**) The oxygen consumption rates of basal respiration, ATP production, proton leak, maximal respiration, spare respiratory capacity and non-mitochondrial respiration of HepG2 cells transfected with miR-141-3p mimics or inhibitor were measured and calculated as averages for each phase. The ATP production was significantly increased in the HepG2 cells transfected with miR-141-3p mimics, while it was markedly decreased in the HepG2 cells transfected with miR-141-3p inhibitor. (**C**,**D**) The expression level of mtDNA-encoded subunits of the OXPHOS complexes was measured. (**C**) The expression level of mRNA (*COX2, COX3, ND1*) involving in OXPHOS was increased markedly in HepG2 cells transfected with miR-141-3p mimics. (**D**) The expression level of mRNA (*COX2, COX3*) was significantly decreased in HepG2 cells transfected with miR-141-3p inhibitor. *P < 0.05.

In order to test whether abnormal express of miR-141-3p will affect the mitochondrial respiratory function, we performed real-time bioenergetic kinetics, using the Seahorse extra cellular analyzer on HepG2 cells, which were transfected with miR-141-3p mimics and inhibitors. The oxygen consumption rates of ATP production were strikingly elevated and reduced in HepG2 cells transfected with miR-141-3p mimics and inhibitors(Fig. 3A,B), respectively. To further investigate the mitochondrial function, we detected the gene expression of mtDNA-encoded OXPHOS subunits. The expression level of several genes (*COX2, COX3, ND1*) involving in OXPHOS elevated significantly in miR-141-3p mimics group (Fig. 3C). The expression level of genes (*COX2, COX3*) was strikingly decreased in miR-141-3p inhibitor group (Fig. 3D). These findings demonstrated that miR-141-3p regulated mitochondrial activity in HepG2 cells.

**Figure 4:**
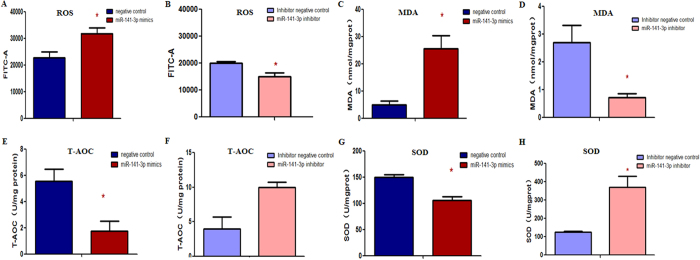
The miR-141-3p may induce oxidative stress in the HepG2 cells. The ROS production (**A**) and MDA content (**C**)were both significantly increased in HepG2 cells transfected with miR-141-3p mimics and both markedly decreased in HepG2 cells transfected with miR-141-3p inhibitor (**B**,**D**). Antioxidant capacity, including T-AOC activities and SOD activities were measured. Both of them were significantly decreased in HepG2 cells transfected with miR-141-3p mimics (**E**,**G**), and increased in HepG2 cells transfected with miR-141-3p inhibitor (**F**,**H**). Each data point represented the mean ± SE from three separate experiments in which treatments were performed in triplicate. *P < 0.05.

To further investigate the oxidative stress of HepG2 cells, we detected both oxidative damage and antioxidant capacity. The ROS and MDA production were found significantly increased in miR-141-3p mimics group (Fig. 4A,C), while both of them were markedly reduced in miR-141-3p inhibitor group (Fig. 4B,D). These indicated that the miR-141-3p was prone to induce oxidative damage. Additionally, both T-AOC and SOD activities were significantly decreased in miR-141-3p mimics group (Fig. 4E,G), while the T-AOC was partly increased and the SOD activity(Fig. 4F,H) was markedly increased in miR-141-3p inhibitor group, suggesting that miR-141-3p compromised the antioxidant defense system. After transfecting miR-141-3p mimics or inhibitors, there was no significant change in the mitochondrial content (Fig. S3A, B) or the concentrations of TC (Fig. S3C, D) triglyceride (TG) (Fig. S3E, F).

To assess the physical condition of HepG2 cells, we detected the mitochondrial membrane potential and cell apoptosis by flow cytometry, and the cell proliferation by CCK8 assay kit. After transfecting miR-141-3p mimics or inhibitors, there was no significant change in the mitochondrial membrane potential (Fig. S4A-F), the cell apoptosis (Fig. S5A-F) nor the cell proliferation (Fig. S5G, H).

MiR-141-3p also involved in regulating pro-inflammatory cytokines. To determine whether the lipid content (FFA), glucose, insulin and pro-inflammatory cytokines (*interleukin 6, IL-6* and *tumor necrosis factor, TNF-α*) could affect miR-141-3p expression, the HepG2 cells was treated with the exogenous stimulation and miR-141-3p expression level was detected. As shown in Fig. S6A, after glucose stimulation in the media, the mitochondria-related miR-141-3p expression level was significantly increased. However, after FFA, insulin, IL-6 or TNF-α stimulation in the media, the mitochondria-related miR-141-3p expression level was higher in the treated groups, although no significant difference was detected. Taken together, exposure of the HepG2 cells to high-glucose resulted in the up-regulation of miR-141-3p expression level, suggesting that miR-141-3p was regulated by high-glucose in HepG2 cells. In combination with result that the higher plasma glucose level in the HFD mice, our data suggested that miR-141-3p might be involved in the obesity-related glucose intolerance. However, the possible mechanism of high-glucose in regulating miR-141-3p expression remains unknown.

To explore the effect of mitochondria-related miR-141-3p on the pro-inflammatory cytokines, we measured the expression of pro-inflammatory cytokines (*IL-6 and TNF-α*) in HepG2 cells, as shown in Fig. S6B, we found that the *IL-6* mRNA expression level was significantly increased in the HepG2 cells transfected with miR-141-3p mimics, and reduced in the HepG2 cells transfected with miR-141-3p inhibitor, however, there was no significant difference of *TNF-α* mRNA expression level in the HepG2 cells. Above findings suggested that the mitochondria-related miR-141-3p might promote the pro-inflammatory cytokine (IL-6) expression, inducing the inflammatory response and contributing to the development of obesity.

### PTEN as a target of miR-141-3p

**Figure 5:**
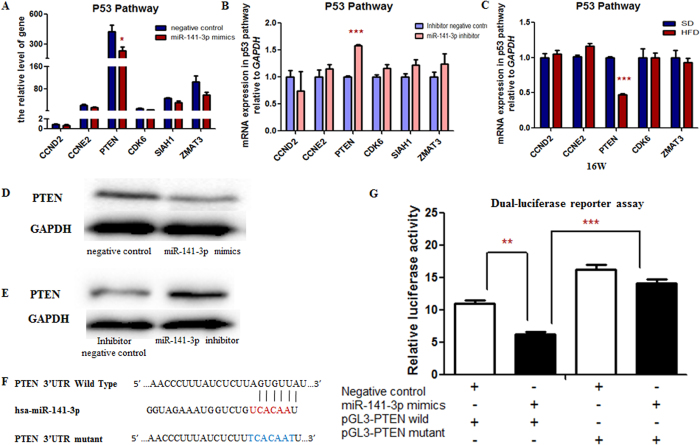
*PTEN* was the target of the mitochondria-related miR-141-3p. The expression level of *PTEN* mRNA in p53 pathway was significantly decreased in the HepG2 cells transfected with miR-141-3p mimics (**A**) while it was significantly increased in the HepG2 cells transfected with miR-141-3p inhibitor (**B**) and in the HFD mice livers (**C**). The representative of western blot of PTEN was shown. The protein level of PTEN was decreased in the HepG2 cells transfected withmiR-141-3p mimics (**D**) while it was increased in the HepG2 cells transfected with miR-141-3p inhibitor (**E**). (**F**) Sequence alignment of mitochondria-related miR-141-3p with 3′UTR of *PTEN*. Bottom: mutations in the 3′UTR of *PTEN*. (**G**) The firefly luciferase activity was normalized with Renilla. Each data point represented the mean ± SE from three separate experiments in which treatments were performed in triplicate. *P < 0.05, **P < 0.01, ***P < 0.001.

To further investigate the mechanism of miR-141-3p regulating mitochondria, relative signaling pathway was analyzed. It’s known that, DNA Intelligent Analysis (DIANA) DIANA-miR Path v2.0 is specifically focused on the identification of miRNA target pathway^10^. With the use of this web server, we found that p53 pathway was associated with miR-141-3p. Expression levels of the target genes (*PTEN, ZMAT3, CCND2, CCNE2, CDK6, SIAH1*) of miR-141-3p that were abundant in p53 pathway were detected, and we found that *PTEN* was significantly down-regulated in miR-141-3p mimics group (Fig. 5A) and up-regulated in miR-141-3p inhibitor group (Fig. 5B). Besides, the *PTEN* mRNA expression in the mice was also detected, as shown in Fig. 5C, the *PTEN* mRNA expression level was significantly decreased in the HFD mice. The protein level of PTEN was also reduced in miR-141-3p mimics group (Fig. 5D) and increased in miR-141-3p inhibitor group (Fig. 5E), respectively.

Then we performed dual-luciferase reporter assay to validate whether miR-141-3p regulated *PTEN* directly by binding to the 3′UTR region of *PTEN*. As the results shown (Fig. 5G), the relative luciferase activity of the HepG2 cells co-transfected with miR-141-3p mimics and pGL3-PTEN wild type was significantly inhibited when compared with the HepG2 cells co-transfected with negative control and pGL3-PTEN wild type. It was also decreased when compared with HepG2 cells co-transfected with miR-141-3p mimics and pGL3-PTEN mutant type. It demonstrated that miR-141-3p regulated *PTEN* by combining the 3′UTR region of *PTEN* mRNA.

**Figure 6:**
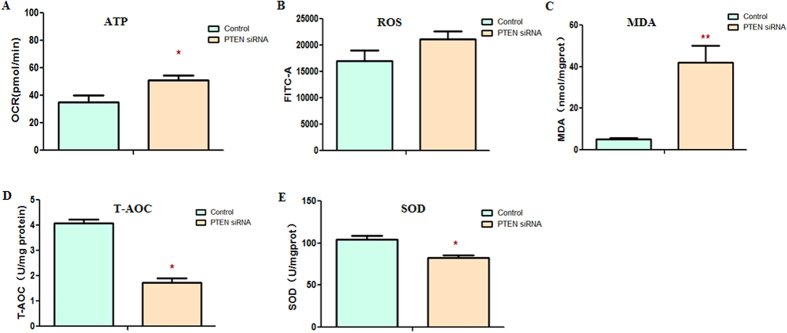
The *PTEN* may involve in the mitochondrial activity of the HepG2 cells. (**A**) The OCR of ATP production was measured in HepG2 cells transfected with PTEN siRNA and it was markedly increased. The ROS production (**B**) and MDA content (**C**) were both increased in the HepG2 cells transfected with PTEN siRNA. While T-AOC (**D**) and SOD activity (**E**) were both significantly reduced in the HepG2 cells transfected with PTEN siRNA. Each data point represented the mean ± SE from three separate experiments in which treatments were performed in triplicate. *P < 0.05.

We have validated that the miR-141-3p interacted with *PTEN* by binding to its 3′UTR region and influenced mitochondrial function of HepG2 cells. To further verify whether miR-141-3p affected mitochondrial function of HepG2 cells through *PTEN* directly, we conducted experiments with HepG2 cells transfected with *PTEN* siRNA. Amazingly, we found that the OCR of ATP production was strikingly increased (Fig. 6A).The ROS production was higher in the PTEN siRNA group without significant difference (Fig. 6B), while the MDA content was significantly increased in PTEN siRNA group (Fig. 6C). Additionally, significant down-regulation of T-AOC (Fig. 6D) and SOD activity (Fig. 6E) were observed in PTEN siRNA group, compared with the control group. Above findings demonstrated that *PTEN* was involved in mitochondrial function.

To measure the transfect efficiency of miR-141-3p mimics and inhibitors, we detected the miR-141-3p expression level in HepG2 cells before and after treatment. As shown in Fig.S6C, the basal expression of miR-141-3p was relatively low in the HepG2 cells. Moreover, the miR-141-3p expression level was significantly up-regulated in the miR-141-3p mimics group and down-regulated in the miR-141-3p inhibitor group (Fig. S6D), respectively. These findings suggested that the miR-141-3p mimics and inhibitor was successfully transfected into the HepG2 cells. Likewise, the *PTEN* mRNA expression level was significantly reduced (Fig. S6E) in HepG2 cells transfected with PTEN siRNA, suggesting that the PTEN siRNA was successfully transfected into the HepG2 cells, too.

## Discussion

In the present study, we found that (i) the expression of miR-141-3p was increased in HFD-induced obese mice liver; (ii) miR-141-3p contributed to the altered mitochondrial function, including up-regulation of ATP production, ROS production, MDA content and down-regulation of T-AOC activity, SOD activity; (iii) by silencing *PTEN*, ATP production was increased and oxidative stress was induced. In conclusion, our study provided novel evidence that the miR-141-3p contributed to mitochondrial dysfunction in HFD-induced obesity.

Obesity, one common metabolic disorder, is the result of excessive energy accumulation in white adipocytes^11^. Accumulating reports suggested that obesity was associated with mitochondrial dysfunction, including abnormalities in mitochondrial fission and fusion^12^, impaired mitochondrial biogenesis^4^, oxidative stress and inflammation^13^,^14^. Recently, mitochondria-related miRNAs have been reported to play vital roles in mitochondrial metabolism, mitochondria-mediated apoptosis, mitochondrial morphology and mitophagy^7^,^9^. It has been reported that the mitochondria-related miR-141-3p was the potential biomarker of various diseases, including primary biliary cirrhosis^15^, hepatocellular carcinoma^16^, bladder cancer^17^and colorectal cancer^18^. However, the role of mitochondria-related miR-141-3p in the development of obesity remains unknown. Thus, it can be hypothesized that mitochondria-related miR-141-3p might contribute to the mitochondrial dysfunction in the development of obesity.

In our study, miR-141-3p promoted the ATP production by targeting *PTEN* in HFD mice livers and hepG2 cells. However, Baseler *et al.*, found that miR-141 could decrease ATP synthase activity by inhibiting its target, *solute carrier family 25 member 3 protein* (*Slc25a3*)^19^. It might be caused by different cell types or tissues. And the expression level of *Slc25a3* was abundant in heart^20^. Recently, Roe *et al.*, demonstrated that loss of *PTEN*, target of miR-141-3p, was able to increase ATP levels^21^. Yang Li *et al.*, validated that *PTEN* loss could induce mitochondrial respiration (OCR)^22^. Thus, in line with the two studies, our results indicated that miR-141-3p over-expression reduced *PTEN* expression and promoted ATP production.

We also showed that the mitochondria-related miR-141-3p increased the expression of *COX2* and *COX3*. The *COX1*, *COX2* and *COX3*, which are the mitochondria-encoded subunits of cytochrome c oxidase (COX), form the catalytic core of enzyme^23^. When the demand for ATP from OXPHOS increased, the expression of COX was up-regulated, too^23^. Collectively, our study demonstrated that mitochondria-related miR-141-3p could promote OXPHOS process and increase ATP production.

Mitochondria are believed to be both the source and target for ROS^24^,^25^. Besides, MDA was generated along with the production of ROS^10^. MDA, an end-product of the breakdown of polyunsaturated fatty acids, is a proper estimate of lipid peroxidation^26^. MDA targets mitochondrial complexes and disrupts flow of electrons through the electron transport chain. While protective actions against ROS are performed by several enzymes (e.g. SOD, catalase and glutathione peroxidase, which are classical antioxidants^25^). T-AOC, one of antioxidant biomarkers, represents the total antioxidant capacity^27^. Collectively, our results of oxidative stress assay suggested that the mitochondria-related miR-141-3p induced the oxidative damage while reduced the antioxidant capacity. The alterations of ATP and ROS production usually associated with the changes in mitochondrial membrane potential, cell apoptosis and cell proliferation^28^, however, these alterations do not always appear simultaneously. In present study, we found that mitochondria-related miR-141-3p had no impacts on mitochondrial membrane potential, cell apoptosis nor cell proliferation. Above all, the mitochondria-related miR-141-3p might contribute to the mitochondrial dysfunction. We also found that the mitochondria-related miR-141-3p might promote the pro-inflammatory cytokine (*IL-6*) expression, inducing the inflammatory response and contributing to the development of obesity.

*PTEN* is a tumor suppressor gene and plays a crucial role in maintaining normal cell activities^29^. Previous studies have verified that *PTEN* over-expression in mice resulted in reduced body weight^30^,^31^. Similarly, *PTEN* haploinsufficiency in human resulted in obesity^32^. All these findings suggested that there was a strong association between decreased *PTEN* and obesity. It was also demonstrated that the loss of *PTEN* contributed to the process of mitochondrial biogenesis, respiration and increased the mitochondrial volume and ROS production by activating the insulin-activated PI3K/AKT pathway, a main anabolic pathway^29^. In our study, by silencing *PTEN*, ATP production was increased and oxidative stress was induced.

In summary, present study first demonstrated that mitochondria-related miR-141-3p suppressed the expression of *PTEN*, resulting in HepG2 cells mitochondrial dysfunction, presumably through the elevation of ATP production, oxidative stress, and the reduction of antioxidant capacity. All these changes led to energy imbalance, and finally the obesity was developed. Our study hopefully improved current understanding of the role of mitochondria-related miRNAs in obesity.

## Materials and Methods

### Animal care

All experiments involving animals and tissue samples were performed in strict accordance with the Care and Use of Laboratory Animals of the National Institues of Health (NIH) and all animal procedures were approved by the Institutional Animal Care and Use committee (IACUC) of Nanjing Medical University (ID: 2011082112). The animals were housed in a polycarbonate cage at Nanjing Medical University in a temperature, humidity-controlled (23 ± 1 ^o^C, 53 ± 2%) room and maintained on a light cycle (12 h/12 h light/dark) with free access to food and water. Seven-week-old male C57BL/6J mice were obtained from Vital River Laboratories (Beijing, China) and acclimated to our animal room with a standard chow diet for 1 week.

At eight-week-old the mice were divided into two groups (n = 10 per group): (a) control mice given a standard diet (SD: 12.1% fat, 23.2% protein, 64.7% carbohydrate by energy; Xietong, China), and (b) HFD mice given a high-fat diet (HFD: 38.4% fat, 11.4% protein, 50.2% carbohydrate by energy; Xietong, China),for 8 weeks. Main composition of energy of HFD and SD was shown in Table S1. Moreover, the detailed composition of HFD was shown in Table S2. Body weight and food intake were measured weekly and every three days, respectively. For the measurement of food intake, pre-weighed food was placed into a container in the clean cage and three days later, the remainder was collected and weighed. Besides, the Lee’s index is calculated as the following formula, Lee’s index = {[body weight (g)]^1/3^} × 1000/ [body length (cm)].

### Measurement of Metabolic Parameters of C57BL/6J mice

#### Analysis of histological features

Liver and WAT samples were fixed in 10% buffered formalin, embedded in paraffin, sectioned and stained with hematoxylin & eosin (H&E).

#### Analysis of serum insulin level and homeostasis model assessment of insulin resistance (HOMA-IR)

Serum insulin level was measured by Enzyme Linked Immunosorbent Assay (ELSA). The analysis was conducted using Mouse Insulin Elisa Kit (Elabscience, China). The homeostasis model assessment of insulin resistance (HOMA- IR) was calculated as described by Hosker *et al.*^33^. HOMA-IR = [fasting insulin (μU/mL) × fasting glucose (mmol/L)]/22.5.

#### Analysis of glucose levels and oral glucose tolerance test (OGTT) and insulin tolerance test (ITT)

OGTT was carried out at sixteen-week-old, after 12 h of fasting, C57 was intragastric administration of a 25% glucose solution (2 g/kg body weight). For ITT after 6 h of food deprivation, insulin (0.5 u/kg body weight) was injected intraperitoneal injection (I.p). For both analyses, blood samples were taken from the tail at the indicated times (every 30 min from 0 min to 120 min) and blood glucose concentrations were measured using Roche glucometer.

#### Analysis of hormone

The serum hormone of TC, TG, HDL-C, LDL-C, ALT, AST were measured with Hitachi 7100 automatic biochemical analyzer. And liver TC and TG levels were measured utilizing Tissue total cholesterol assay kit (Applygen Technologies Co. Ltd., China) and Tissue triglyceride assay kit (Applygen Technologies Co. Ltd., China), respectively.

### Measurement of mitochondrial function of C57BL/6J mice

#### Analysis of Oxidative Stress of liver

The SOD, T-AOC and MDA in livers were all measured spectrophotometrically using commercially available assay kits (Jiancheng, China), in line with the manufacturer’s protocol^34^. Level of T-AOC, one of the products of lipid peroxidation, was measured via Fe^3+^ disoxidation method^26^, SOD activity was determined according to xanthine oxidase method^35^, and MDA was assayed in line with Thibabituric Acid-Reactive Substance assay (TBARS). Values were normalized to protein concentrations using the Total protein quantitative assay kit (Jiancheng, China). The ROS was measured as followed, fresh livers were added with 1.5 ml 2% FBS-DSB (2 ml FBS mixed with 98 ml PBS), and ground up. After being filtering, the cell suspension was centrifuged at 12 000 rpm for 10 min, and the supernatant was discarded. 1 ml 2% FBS-DSB was added to prepare single-cell suspension, then the single-cell suspension was incubated with 1 μl 1 μg/ml oxidation sensitive probe DCFH-DA (Sigma-aldrich, MO, USA) at 37 °C without light. Finally, the cellular fluorescence intensity was measured with FACS Calibur Flow Cytometry (BD Biosciences, NJ, USA). All the oxidative stress experiment were performed when the C57BL/6J mice were sixteen-week-old.

#### Analysis of ATP of liver

The liver ATP levels were measured when the C57BL/6J mice were sixteen-week-old, using a firefly luciferase ATP assay kit (Beyotime, China) according to the manufacturer’s instructions. 1 ml lysis buffer was added to lysis 100 mg liver and each sample was collected and centrifuged at 12 000 rpm for 5 min at 4 °C. The supernatant was transferred to a new tube for the ATP test. The luminescence from a 100 mL sample was assayed in a 96-well plate luminometer (Berthold Detection System, Pforzheim, Germany) together with 100 mL ATP detection buffer from the ATP detection kit.

#### Analysis of mitochondrial Copy Numberof liver

At sixteen-week-old, the mitochondrial copy number of liver was measured. The quantification of mtDNA was accomplished by calculating the ratio of a mitochondrion-encoded gene (*16s rRNA*) to a nuclear-encoded gene (*Hexokinase* 2), and expressing it as mtDNA copy number per animal. Primer sequences are shown in Table S3.

### Selection of mitochondria-related miRNAs

To date, it has been reported that many mitochondria-related miRNAs affect mitochondrial energy metabolism^6^,^9^. In order to identify mitochondria related miRNAs, we reviewed related studies which reported that some miRNAs might play critical roles in the maintenance of mitochondria function. These mitochondria related miRNAs were involved in the ATP production, mitochondrial metabolism, mitochondrial ROS, mitochondria dynamics, mitophagy, apoptosis or mitochondrial Ca^2+^ homeostasis, respectively. A detailed list of these mitochondria related miRNAs has been shown in Table S4^36^,^37^,^38^,^39^,^40^,^41^,^42^,^43^,^44^,^45^,^46^,^47^,^48^,^49^,^50^,^51^,^52^,^53^,^54^,^55^,^56^,^57^,^58^,^59^,^60^,^61^,^62^,^63^,^64^,^65^.

In the following study, we used HepG2 cells, which are human hepatocellular carcinoma cells, to study the miRNA and mitochondrial function, so we first compared the conservation of these miRNAs between human and mice. Then, based on the mirbase website, 7 miRNAs (including miR-141-3p, miR-196a-5p, miR-499a-5p, miR-126a-3p, miR-210-3p, miR-378a-3p and miR-484), which had conserved sequence in both human and mouse, were selected to investigate their roles in the obesity.

### Measurement of mitochondria-related miRNAs

The quantitative RT-PCR was applied to measure the expression of mitochondria-related miRNAs in the livers of 16-week-old mice. RNA was isolated with the use of TRIZOL reagent (Invitrogen, Carlsbad, CA) following the manufacturer’s instructions, and the concentration of total RNA was determined by measuring the absorbance at 260 nm using a NanoDrop 2000 (Thermo Fisher Scienti c, Wilmington, DE). Real-time PCR was conducted with SYBR Green PCR Master Mix (Takara, Japan). All experiments were repeated three times. Primer sequences are shown in Table S3.

### Cell culture, transfection and treatment

HepG2 cells (ATCC, HB-8065) were obtained from ATCC (Manassas, VA, USA) and cultured in complete growth medium DMEM (Gibco, USA), supplemented with 10% fetal bovine serum(FBS) (Gibco, USA), 100 U mL^−1^penicillin (Gibco, USA), and 100 μgmL^−1^streptomycin (Gibco, USA) at 37 °C, 5% CO_2_. The cells were fed every 2–3 days.

MiR-141-3p mimics, mimics negative control, miR-141-3p inhibitor, inhibitor negative control and siRNA of PTEN oligonucleotide (Genepharm, China) were transiently transfected into HepG2 cells with the use of Lipofectamine 2000 (Invitrogen, CA, USA) according to the manufacturer’s instructions. Cells were washed with PBS and collected with trypsin/EDTA for experiments after 24 h of transfection.

Before treatment, HepG2 cells were seeded in 24-well plate. When ~80% confluence was reached, the cells were incubated in FBS-free medium for 24 hours, then the cells treated with FFA(100 mM)^66^, TNF-α (10 ng/ml)(Sigma)^67^, IL-6 (30 ng/ml)(Sigma)^68^, insulin(50 μU/ml)^69^, glucose(5 mg/ml)^69^, respectively.

### Dual-luciferase reporter assay

To validate whether or not miR-141-3p regulated *PTEN* by binding to the 3’UTR region of *PTEN* mRNA, the dual-luciferase reporter assay was performed. The sequence of 3’UTR region of *PTEN* predicted to interact with miR-141-3p was inserted into the XbaI site of pGL3 promoter vector (Generay, China). These constructs were named as pGL3-*PTEN* wild and pGL3-*PTEN*-mut, respectively.

Twenty-four hours after transfection, cells were collected to measure the luciferase activity with a Dual-Luciferase Reporter Assay System (Promega, WI) and the pRL-SV40 plasmids were used as normalizing control. All experiments were performed in triplicate independently.

### Measurement of mitochondrial function of HepG2 cells

#### Analysis of mitochondrial oxygen consumption and extra cellular flux of HepG2 cells

Cells were seeded to 4,000 cells/well in XF96-well cell culture microplate (Seahorse Bioscience) with 80 μl complete growth medium DMEM. Seahorse Bioscience XF96 Extra cellular Flux Analyzer was used (Seahorse Bioscience, North Billerica, MA, USA) to monitor the OCR in intact HepG2 cells. OCR was measured using mix/wait/measure times of 3/2/4 min. After baseline measurements of OCR, OCR was measured after sequentially adding to each well oligomycin (1.5 μM), carbonyl cyanide-ptrifluoromethoxyphenylhydrazone (FCCP) (0.5 μM), and Rotenone/antimycin A (0.5 μM) to the indicated final concentrations using the included ports on the XF96 cartridges. Further analysis of these experiments was performed as described^25^.

#### Analysis of Oxidative Stress of HepG2 cells

The methods of HepG2 cells were the same with that of animal.

#### Analysis of Mitochondrial Copy Number of HepG2 cells

Quantification of mtDNA was accomplished by calculating the ratio of a mitochondrion-encoded gene *(COX1*) to a nuclear-encoded gene (*28sRNA*), and expressing it as mtDNA copy number per cell. Primer sequences are shown in Table S3.

#### Analysis of mRNA levels of mtDNA-encoded subunits of the OXPHOS complexes, p53 pathway genes and pro-inflammatory cytokines of HepG2 cells

The mRNA levels for mtDNA-encoded OXPHOS subunits, p53 pathway were analyzed using SYBR PCR Master Mix reagent kits (Takara, Tokyo, Japan) according to the manufacturer’s instructions. All oligo nucleotide primers were synthesized by Invitrogen (Shanghai, China). All real-time PCR reactions were carried out on an ABI7900 Fast Real-Time System (Applied Bio systems, Foster City, CA, USA) according to the manufacturer’s instructions. All experiments were repeated at least three times. Primer sequences are shown in Table S3.

#### Analysis of protein level of PTEN of HepG2 cells

The total cellular proteins (60 μg) were solubilized in the sample buffer and then fractionated by electrophoresis on a 10% polyacrylamide-SDS gel at 60 V for 3 h. The proteins were then transferred to a polyvinylidenedifluoride membrane (PVDF, Bio-Rad, Hercules, CA). The membrane with transferred proteins was incubated in buffer containing specific rabbit polyclonal antibodies for PTEN (Abcam, Kendall square, MA, USA, 1:1,000 dilution), followed by incubation with goat anti-rabbit, a secondary antibody conjugated with horsera culture plates peroxidase at 1:1,000. The specific signals were detected by the enhanced chemiluminescence (ECL Western blotting detection reagents, Amersham Life Science Limited). The amount of GAPDH (34 kDa) in each lane was used as a control to correct the expression of PTEN protein (47 kDa).

#### Analysis of mitochondrial membrane potential of HepG2 cells

The cells were incubated with 2 mL of medium containing JC-1 staining probe for 20 min at 37 °C and washed once with staining buffer. Some cells were suspended in 1 mL of PBS and analyzed by flow cytometry to detect green and red fluorescence. The wavelengths of excitation and emission were 488 nm and 530 nm, respectively.

#### Analysis of cell proliferation and cell apoptosis of HepG2 cells

The cell proliferation was measured by using the CCK-8 cell counting kit (Vazyme, China). The absorbance was detected at 450 nm.

Cells were washed with cold PBS, stained with propidium iodide (PI) and annexin V for 30 min protected from light. The fixed/stained cells were analyzed by FACS Calibur Flow Cytometry (BD Biosciences, NJ, USA) to quantify the cell apoptosis.

### Statistical Analysis

Results are expressed as means ± standard error (SE). Statistical analyses were performed using STATA11 and presented with GraphPAD prism software (San Diego, CA). One-way analysis of variance (ANOVA), rank sum test and student’s t test were used to compare quantitative data among groups.

## Additional Information

**How to cite this article**: Ji, J. *et al.* Mitochondria-related miR-141-3p contributes to mitochondrial dysfunction in HFD-induced obesity by inhibiting *PTEN*. *Sci. Rep.* **5**, 16262; doi: 10.1038/srep16262 (2015).

## Supplementary Material

Supplementary Information
