# Supplementary material for: Mitochondria-related miR-141-3p contributes to mitochondrial dysfunction in HFD-induced obesity by inhibiting PTEN
Source: Sci Rep. 2015 Nov 9;5:16262. doi: 10.1038/srep16262 (PMC4637860; doi:10.1038/srep16262)
Supplement: Supplementary Information [file srep16262-s1.doc]

**Mitochondria-related miR-141-3p contributes to mitochondrial dysfunction in HFD-induced obesity by inhibiting *PTEN***

**Juan Ji^1, 2,†^, Yufeng Qin^1, 2,†^, Jing Ren^1, 2,†^, Chuncheng Lu^1, 2,*^, Rong Wang^4^, Xiuliang Dai^4^, Ran Zhou^1, 2^, Zhenyao Huang^1, 2^, Miaofei Xu^1, 2^, Minjian Chen^1, 2^, Wei Wu^1, 2^, Ling Song^1, 2^, Hongbing Shen^1, 3^, Zhibin Hu^1, 3^,** **Dengshun Miao^1, 4^**, **Yankai Xia^1, 2^, and Xinru Wang^1, 2,*^**

^1^ State Key Laboratory of Reproductive Medicine, Institute of Toxicology, Nanjing Medical University, Nanjing 210029, China

^2^ Key Laboratory of Modern Toxicology of Ministry of Education, School of Public Health, Nanjing Medical University, Nanjing 210029, China.

^3^ Department of Epidemiology and Biostatistics and Key Laboratory of Modern Toxicology of Ministry of Education, School of Public Health, Nanjing Medical University, Nanjing, China.

^4^ Research Center for Bone and Stem Cells, Department of Anatomy, Histology, and Embryology, Nanjing Medical University, Nanjing, China.

**^†^** The authors have contributed equally to this study and they should be regarded as joint first authors.

^*^ To whom correspondence should be addressed at:

Drs. Chuncheng Lu and Xinru Wang

State Key Laboratory of Reproductive Medicine, Institute of Toxicology, Nanjing Medical University, Nanjing 210029, China

Phone: +86-25-86862845; Fax: +86-25-86862847

E-mail: chunchenglu@njmu.edu.cn and [xrwang@njmu.edu.cn](mailto:xrwang@njmu.edu.cn)

Table S1. Main composition of energy of high-fat-diet and standard diet

|  | **HFD** | **SD** |
| --- | --- | --- |
|  | **Kcal/Kg Energy (%)** | **Kcal/Kg Energy (%)** |
| **Protein**  **Fat**  **Carbohydrate** | 552 11.4  1854 38.4  2424 50.2 | 792 23.2  413 12.1  2209 64.7 |

Table S2. Detailed composition of high-fat-diet

| **Component** | **percentage (%)** |
| --- | --- |
| **Commercial non-purified diet**  **Lard**  **Sesam oil**  **Glucose**  **Cholesterol**  **Sodium cholate**  **Peanut** | 56.9  13  2  20  3  0.1  5 |

Table S3. Sequences of primers for RT-PCR

| ***miRNA name*** | ***RT, Forward and Reverse primer (5’-3’)*** | |
| --- | --- | --- |
| U6 | RT: CTCAACTGGTGTCGTGGA | |
|  | F: CTCGCTTCGGCAGCACA | R: AACGCTTCACGAATTTGCGT |
| mmu-miR-141-3p | RT:GTCGTATCCAGTGCAGGGTCCGAGGTATTCGCACTGGATACGACCCATCT | |
|  | F:GCGGCGGTAACACTGTCTGG | R:AACGCTTCACGAATTTGCGT |
| mmu- miR-196a-5p | RT:GTCGTATCCAGTGCAGGGTCCGAGGTATTCGCACTGGATACGACCCCAAC | |
|  | F: GCGGCGGTAGGTAGTTTCATGTTG | R: AACGCTTCACGAATTTGCGT |
| mmu- miR-484 | RT:GTCGTATCCAGTGCAGGGTCCGAGGTATTCGCACTGGATACGACATCGGG | |
|  | F: GCGGCGGTCAGGCTCAGTCCCC | R: AACGCTTCACGAATTTGCGT |
| mmu- miR-499-5p | RT:GTCGTATCCAGTGCAGGGTCCGAGGTATTCGCACTGGATACGACAAACAT | |
|  | F: GCGGCGGTTAAGACTTGCAGTG | R: AACGCTTCACGAATTTGCGT |
| mmu- miR-210-3p | RT:GTCGTATCCAGTGCAGGGTCCGAGGTATTCGCACTGGATACGACTCAGCC | |
|  | F: GCGGCGGCTGTGCGTGTGACAG | R: AACGCTTCACGAATTTGCGT |
| mmu- miR-378a-3p | RT:GTCGTATCCAGTGCAGGGTCCGAGGTATTCGCACTGGATACGACCCTTCT | |
|  | F: GCGGCGGACTGGACTTGGAGTC | R: AACGCTTCACGAATTTGCGT |
| mmu- miR-126a-3p | RT:GTCGTATCCAGTGCAGGGTCCGAGGTATTCGCACTGGATACGACCGCATT | |
|  | F: GCGGCGGTCGTACCGTGAGTAA | R: AACGCTTCACGAATTTGCGT |
| ***Gene name*** | ***Forward and Reverse primer (5’-3’)*** | |
| *mmu-GAPDH* | F: AGGTCGGTGTGAACGGATTTG | R:GGGGTCGTTGATGGCAACA |
| *mmu-16S rRNA* | F:CCGCAAGGGAAAGATGAAAGAC | R: TCGTTTGGTTTCGGGGTTTC |
| *mmu-Hexokinase2* | F:GCCAGCCTCTCCTGATTTTAGTGT | R: GGGAACACAAAAGACCTCTTCTGG |
| *hsa-COX1* | F:CAAACCTACGCCAAAATCCA | R:GAAATGAATGAGCCTACAGA |
| *hsa-28S rRNA* | F:AGGACCCGAAAGATGGTGAACTA | R:CGGAGGGAACCAGCTACTAGAT |
| *hsa-GAPDH* | F:GGATCTCGCTCCTGGAAGATG | R: GGGAACACAAAAGACCTCTTCTGG |
| *hsa-CCND2* | F: ACCTTCCGCAGTGCTCCTA | R:CCCAGCCAAGAAACGGTCC |
| *hsa-CCNE2* | F:TCAAGACGAAGTAGCCGTTTAC | R: TGACATCCTGGGTAGTTTTCCTC |
| *hsa-CDK6* | F: GCTGACCAGCAGTACGAATG | R: GCACACATCAAACAACCTGACC |
| *hsa-SIAH1* | F:AGCCGTCAGACTGCTACAG | R:AAAAGACTCGCCAAGTCATTGT |
| *hsa- ZMAT3* | F:AGAAGCCTTTTGGGCAGGAG | R:TGCTGCATAGTAATTTCGGAGTT |
| *hsa-PTEN* | F:TGGATTCGACTTAGACTTGACCT | R:GGTGGGTTATGGTCTTCAAAAGG |
| *mmu-CCND2* | F: GAGTGGGAACTGGTAGTGTTG | R: CGCACAGAGCGATGAAGGT |
| *mmu-CCNDE2* | F: ATGTCAAGACGCAGCCGTTTA | R: GCTGATTCCTCCAGACAGTACA |
| *mmu-PTEN* | F: TGGATTCGACTTAGACTTGACCT | R: GCGGTGTCATAATGTCTCTCAG |
| *mmu-CDK6* | F: TCTCACAGAGTAGTGCATCGT | R: CGAGGTAAGGGCCATCTGAAAA |
| *mmu-ZMAT3* | F: TGGCTTCCTTTACCTAATCGGC | R: GGGGCTTACACAGCTCCTC |
| *β -actin* | F:ATGGAGCCGGACAGAAAAGC | R:CTTGCCACTCAGGGAAGGA |
| *ND1* | F:TCCGAGCATCTTATCCACG | R:GTATGGTGGTACTCCCGCTG |
| *ND2* | F:ATCCTCCTGGCCATCGTACT | R:ATCAGAAGTGGAATGGGGCG |
| *ND3* | F:TTGCATTCTGACTCCCCCAA | R:GACGTGCAGAGCTTGTAGGG |
| *ND4* | F:TAATCGCACATGGCCTCACA | R:GCTGTGGATCCGTTCGTAGT |
| *ND5* | F:TAACCGCATCGGAGACATCG | R:GTGGAGGCCAAATTGTGCTG |
| *ND6* | F:GGATTGGGGTAGCGGCAATA | R:CCGCAAACAAAGATCACCCA |
| *CYTB* | F:ACGCAAACGGAGCCTCAATA | R:CCTCATGGAAGGACGTAGCC |
| *COX1* | F:GCTAGCCGCAGGCATTACTA | R:CTCCTCCAGCGGGATCAAAG |
| *COX2* | F:AACCGAGTCGTTCTGCCAAT | R:CTAGGGAGGGGACTGCTCAT |
| *COX3* | F:AGCCTTTTCAGCCCTCCTTC | R:GTGGCCTTGGTAGGTTCCTT |
| *ATP6* | F:GCAGTCCGGCTTACAGCTAA | R:GGTAGCTGTTGGTGGGCTAA |
| *ATP8* | F:ACAAACATTCCCACTGGCAC | R:TTGGGGTAATGAATGAGGCAA |
| *IL-6* | F:ACTCACCTCTTCAGAACGAATTG | R:CCATCTTTGGAAGGTTCAGGTTG |
| *TNFα* | F: CCTCTCTCTAATCAGCCCTCTG | R:GAGGACCTGGGAGTAGATGAG |

Table S4. MiRNAs involved in mitochondrial function.

| **MiRNAs name** | **Target** | **Reference** |
| --- | --- | --- |
| **ATP level** | | |
| miR-15/miR-16 miR-195/miR-424 | ARL2 | 36 |
| miR-133 | Prdm16 | 37 |
| miR-141 | Slc25a3 | 18 |
| miR-338 | COX IV | 38 |
| miR-196a | Hoxc8 | 39 |
| **Mitochondrial metabolism** | | |
| miR-23a/b | GLS | 40 |
| miR-210 | ETC components | 41 |
| miR-378 | PGC1β | 42 |
| miR-126 | IRS-1 | 43 |
| miR-199a | PPARδ | 44 |
| **Mitochondrial ROS** | | |
| miR-106b | Mfn2 | 45 |
| miR-145 | Bnip3 | 46 |
| miR-335/miR-34a | SOD2/Txnrd2 | 47 |
| miR-181 | Bcl-2 | 48 |
| **Mitochondria dynamics** | | |
| miR-30 | Mitochondrial fusion | 49 |
| miR-494 | Mitochondrial biogenesis | 50 |
| miR-135b | MID1/MTCH2 | 51 |
| miR-484 | Fis1 | 52 |
| miR-499 | Drp1 | 53 |
| miR-761 | MFF | 54 |
| **Mitophagy** | | |
| miR-101 | - | 55 |
| miR-204 | - | 56 |
| miR-30a | - | 57 |
| miR-21 | PINK1 | 58 |
| **Apoptosis** | | |
| miR-15a/miR-16-1 | Bcl-2/Mcl-1 | 59 |
| miR-210 | Bcl-2 | 60 |
| miR-1 | Bcl-2 | 61 |
| miR-195 | Bcl-2 | 62 |
| miR-365 | Bcl-2 | 63 |
| miR-24 | Bcl-2/XIAP | 64 |
| **Mitochondrial Ca^2+^ homeostasis** | | |
| miR-25 | MCU | 65 |


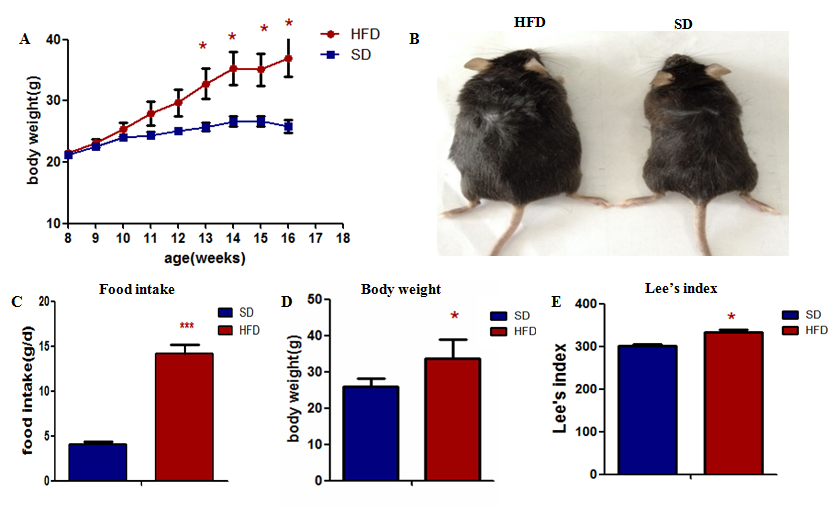


**Fig. S1** The obese model of C57 was successfully established. (A) Body weight curves of SD and HFD mice over 8-week period. Body weight was significantly increased in HFD (vs.SD) from 13-week-old. (B) Spontaneous obesity in a HFD mouse compared with SD was photographed. (C) Daily food intake of both groups during the 8 weeks. The food intake of HFD mice was significantly higher than SD mice. At the age of 16 weeks, The HFD mice gained more body weight (D), larger Lee’s index (E). **P*<0.05.


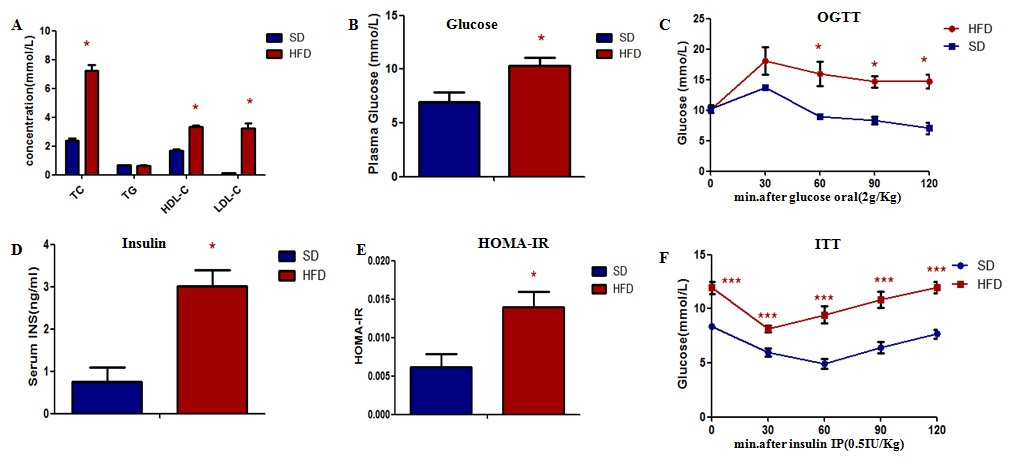


**Fig. S2** HFD mice developed glucose intolerance and insulin resistance. At 16-week-old, serum TC, TG, HDL-C, LDL-C (A), was measured. After 12h fasting, plasma glucose (B) and serum insulin (D) were measured, and HOMA-IR (E) was calculated, the three parameters related with insulin sensitivity were all significantly increased in HFD mice. Glucose homeostasis was monitored by (C) OGTT and (F) ITT, the blood glucose levels of HFD significantly increased. **P*<0.05, ****P*<0.001.


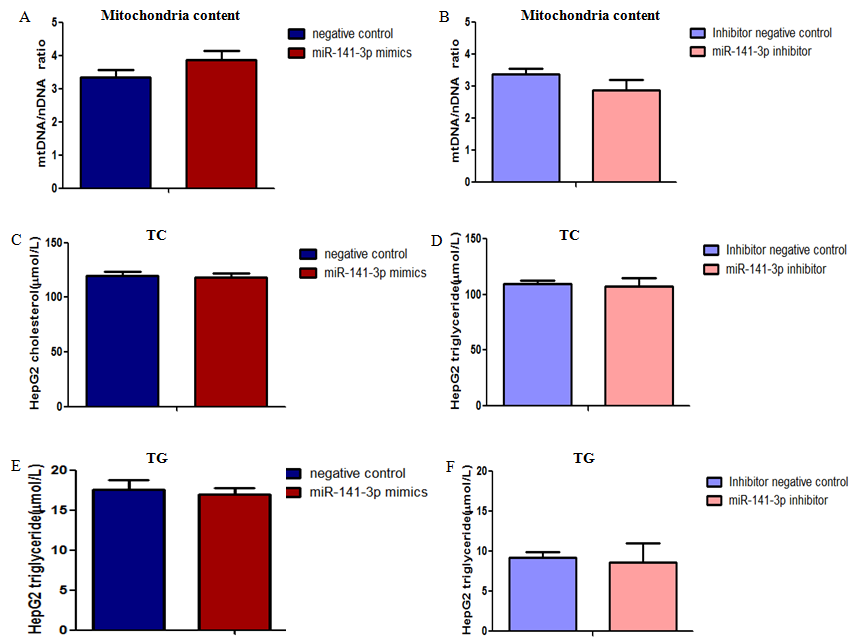


**Fig. S3** The mitochondrial content (A, B), TC (C, D) and TG (E, F) in HepG2 were similar between the negative control group and miR-141-3p mimics group or between the inhibitor negative control group and miR-141-3p inhibitor group. Each data point represented the mean ± SE from three separate experiments in which treatments were performed in triplicate.


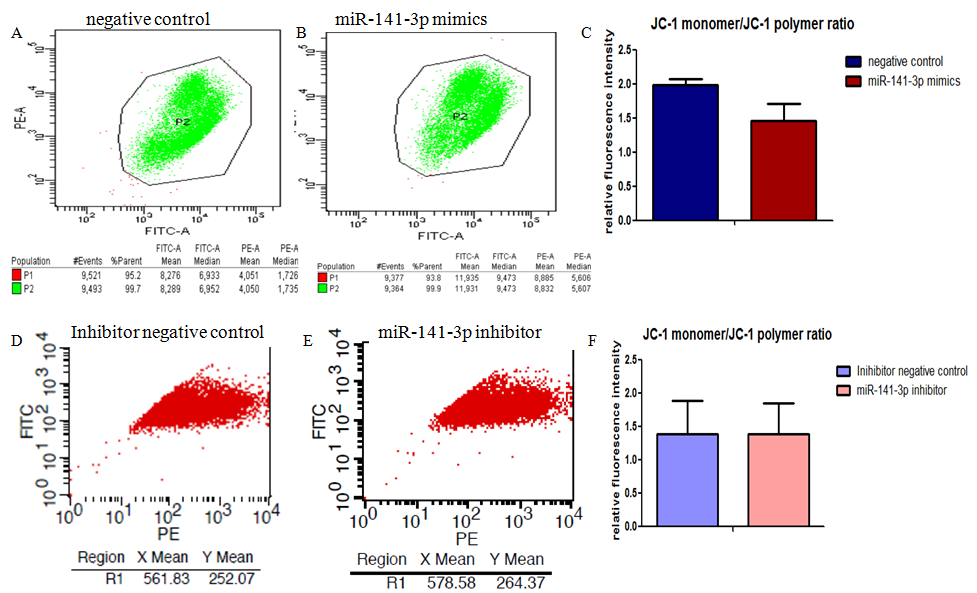


**Fig. S4** (A, B, D, E) Assessment of mitochondrial membrane potential by JC-1 staining and detection by flow cytometry were performed. (C, F) The flow cytometry results of JC-1 staining were presented in a histogram. There was no significant difference was found in ratio of monomer/aggregate between the two groups. Each data point represented the mean ± SE from three separate experiments in which treatments were performed in triplicate.


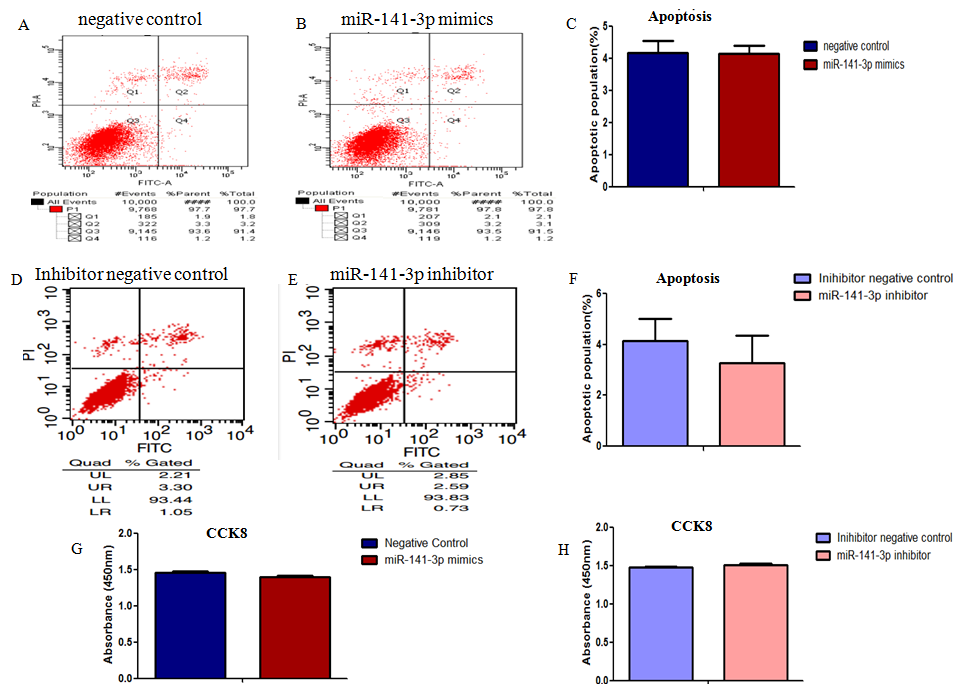


**Fig. S5** (A, B, D, E) Cells in the Q3 quadrant indicated that they were live cells. Cells in the Q4 quadrant were in the early stages of apoptosis. Cells in the Q2 quadrant were late apoptotic. (C, F) The percentage of apoptotic cells (Q2+ Q4) was presented in histogram, there was no significant difference in the apoptosis. (G, H) The cell proliferation was measured by using CCK-8 cell proliferation assay kit. There was no significant difference in the cell proliferation. Each data point represented the mean ± SE from three separate experiments in which treatments were performed in triplicate.


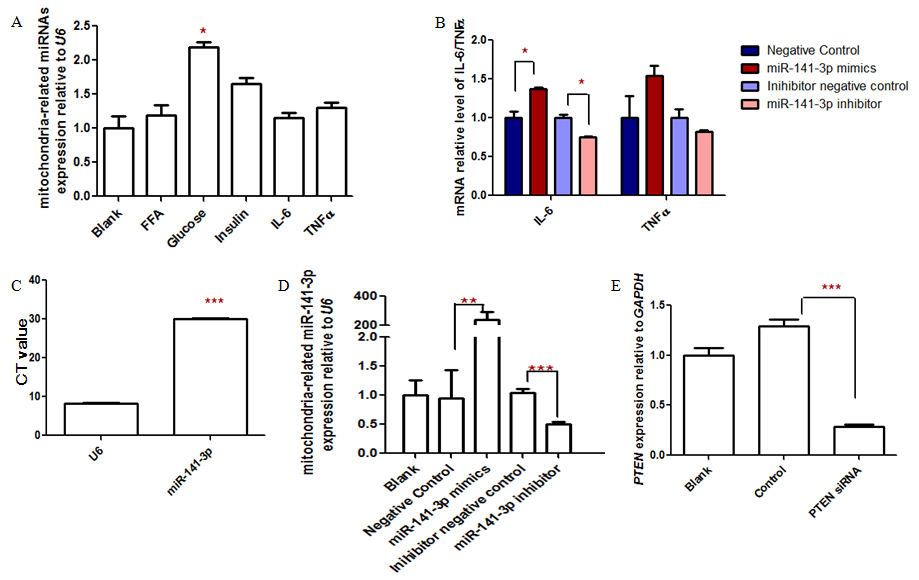


**Fig. S6 (A)** Effect of exogenous stimulation on the miR-141-3p expression level in HepG2 cell line. Cells were treated with the exogenous stimulation and the miR-141-3p expression level was measured. (B) Effect of miR-141-3p on the pro-inflammatory cytokines expression level in HepG2 cells. (C) The basal expression level of miR-141-3p in the HepG2 cells. Transfection efficiency of miR-141-3p mimics, inhibitor (D) and PTEN siRNA (E) into HepG2 cell line*.* Each data point represented the mean ± SE from three separate experiments in which treatments were performed in triplicate. **P*<0.05, ****P*<0.001.
